# Minimally Invasive Ponto Surgery compared to the linear incision technique without soft tissue reduction for bone conduction hearing implants: study protocol for a randomized controlled trial

**DOI:** 10.1186/s13063-016-1662-0

**Published:** 2016-11-09

**Authors:** Tim G. A. Calon, Marc van Hoof, Herbert van den Berge, Arthur J. G. de Bruijn, Joost van Tongeren, Janny R. Hof, Jan Wouter Brunings, Sofia Jonhede, Lucien J. C. Anteunis, Miranda Janssen, Manuela A. Joore, Marcus Holmberg, Martin L. Johansson, Robert J. Stokroos

**Affiliations:** 1 Department of Otorhinolaryngology, Head and Neck Surgery, Maastricht University Medical Center, Maastricht, The Netherlands; 2 Department of Otorhinolaryngology, Medisch Centrum Leeuwarden, Leeuwarden, The Netherlands; 3 Department of Otorhinolaryngology, ZiekenhuisGroep Twente, Almelo, The Netherlands; 4 Oticon Medical AB, Askim, Sweden; 5 Department of Methodology and Statistics, School for Public Health and Primary Care (CAPHRI), Maastricht University, Maastricht, The Netherlands; 6 Department of Clinical Epidemiology and Medical Technology Assessment (KEMTA), Maastricht University Medical Centre, Maastricht, The Netherlands; 7 Department of Biomaterials, Institute of Clinical Sciences, Sahlgrenska Academy, University of Gothenburg, Gothenburg, Sweden

**Keywords:** MIPS, Bone conduction hearing implant (BCHI), Soft tissue preservation, BAHA, Bone conduction device (BCD), Randomized Controlled Trial (RCT)

## Abstract

**Background:**

Over the last years, less invasive surgical techniques with soft tissue preservation for bone conduction hearing implants (BCHI) have been introduced such as the linear incision technique combined with a punch. Results using this technique seem favorable in terms of rate of peri-abutment dermatitis (PAD), esthetics, and preservation of skin sensibility. Recently, a new standardized surgical technique for BCHI placement, the Minimally Invasive Ponto Surgery (MIPS) technique has been developed by Oticon Medical AB (Askim, Sweden). This technique aims to standardize surgery by using a novel surgical instrumentation kit and minimize soft tissue trauma.

**Methods:**

A multicenter randomized controlled trial is designed to compare the MIPS technique to the linear incision technique with soft tissue preservation. The primary investigation center is Maastricht University Medical Center. Sixty-two participants will be included with a 2-year follow-up period. Parameters are introduced to quantify factors such as loss of skin sensibility, dehiscence of the skin next to the abutment, skin overgrowth, and cosmetic results. A new type of sampling method is incorporated to aid in the estimation of complications. To gain further understanding of PAD, swabs and skin biopsies are collected during follow-up visits for evaluation of the bacterial profile and inflammatory cytokine expression.

The primary objective of the study is to compare the incidence of PAD during the first 3 months after BCHI placement. Secondary objectives include the assessment of parameters related to surgery, wound healing, pain, loss of sensibility of the skin around the implant, implant extrusion rate, implant stability measurements, dehiscence of the skin next to the abutment, and esthetic appeal. Tertiary objectives include assessment of other factors related to PAD and a health economic evaluation.

**Discussion:**

This is the first trial to compare the recently developed MIPS technique to the linear incision technique with soft tissue preservation for BCHI surgery. Newly introduced parameters and sampling method will aid in the prediction of results and complications after BCHI placement.

**Trial registration:**

Registered at the CCMO register in the Netherlands on 24 November 2014: NL50072.068.14. Retrospectively registered on 21 April 2015 at ClinicalTrials.gov: NCT02438618. This trial is sponsored by Oticon Medical AB.

**Electronic supplementary material:**

The online version of this article (doi:10.1186/s13063-016-1662-0) contains supplementary material, which is available to authorized users.

## Background

The World Health Organization estimated that approximately 360 million people worldwide suffer from disabling hearing loss (HL) [1]. People with HL can often benefit from the use of hearing devices, such as hearing aids, but patients with, e.g., conductive hearing loss cannot always profit from traditional hearing aids. In order to improve hearing for this group of patients, the bone-anchored hearing aid (BAHA), also known as the bone conduction hearing implant (BCHI) was introduced in 1981 [2]. The BCHI consists of a titanium fixture implanted in the retroauricular bone of the skull with an abutment that breaches the skin, so that a sound processor can be attached to it. The sound processor converts sound waves into vibrations via its transducer. These vibrations are conducted by the abutment to the titanium fixture and ultimately to the skull. The skull conducts these vibrations to both inner ears, bypassing any problems in the ear canal or middle ear [3]. BCHIs are currently considered a suitable treatment option for three groups of patients, namely patients with conductive HL, mixed HL or single-sided deafness (SSD).

This technology has been reported to improve the quality of life for these patients, although the available data is limited [4, 5]. Recently, measuring quality of life and wellbeing using the concept of capabilities has gained more interest [6]. It can be expected that interventions that entail placing a percutaneous implant to improve hearing, may influence an individual in more ways than just solely altering hearing (e.g., the ability to participate in society versus the perceived disadvantages of these types of implants such as cosmetic and social concerns [7, 8]). This perspective will be considered in this trial as well. Over the years, the BCHI has become an established treatment option with approximately 200,000 BCHI surgeries worldwide to date [9].

Although successful, the BCHI has complications. Known problems include skin inflammation, also known as peri-abutment dermatitis (PAD), pain, numbness of the skin around the implant, skin overgrowth, and implant loss [10]. PAD is graded on a five-point scale called the Holgers Index [11] and is perceived as the most common complication of BCHI use with an estimated occurrence of 16.1–38.1 % among all recipients [10]. PAD is an inflammatory process that is presumed to be multifactorial and little is known about the precise etiology of this condition [12]. Shear stress on the skin around the abutment [2, 13] sets on as the implant and abutment combination fixed to the skull is immovable. This may cause tearing of the skin (e.g., while turning on a pillow at night or during head and jaw movements). The implant and abutment are made of titanium, which may elicit a foreign body response [12, 14]. Also the formation of a biofilm on the abutment by bacteria that colonize the wound site due to persistent breach of the skin might play an important role [15, 16]. Another side effect of the BCHI is pain. This is frequently experienced by patients [17], however the exact cause for this, sometimes chronic pain, remains unknown [18–20]. Another condition which has anecdotally been observed is skin sagging, the presence of excess skin cranially to the abutment [21]. This might be problematic as it can influence the sound processor coupling and the proper function of the processor.

Various strategies for reducing complication rates with BCHIs have been employed throughout the years. Traditionally, the skin surrounding the abutment was thinned, but van de Berg et al. noted lower complication rates after BCHI surgery with a less invasive approach [20]. Since then, an even less invasive, single-stage procedure, where the soft tissue surrounding the abutment is left intact [22–25], has become one of the most common techniques used for BCHI surgery. Advantages of this tissue-preserving approach include: less surgical procedure time, reduced numbness or pain and better cosmetic results [22, 24, 25].

The osteotomy preparation, assuring minimal trauma to the bone, space to maneuver the burr head, and adequate cooling of the site as well as minimal trauma and displacement of the soft tissue, are all important factors when designing a procedure for installing a bone-anchored percutaneous implant [26]. Furthermore, to achieve comparable results across surgeries, the variation in surgical technique introduced by different surgeons needs to be minimized. Based on local clinical practices and available tools, surgeons in different countries have started using punch-only surgical techniques for BCHI surgery [27–29]. The available surgical tools were not developed for this punch-only approach, presenting potential drawbacks such as soft tissue damage and insufficient irrigation. In pursuance of developing a standardized minimally invasive punch-only surgical kit and method, Oticon Medical AB (Askim, Sweden) started the design of instruments for a single-stage BCHI surgical technique in 2013. The goal of this new Minimally Invasive Ponto Surgery (MIPS) was to optimize tissue preservation, minimize tissue trauma, and provide a punch-only standardized surgical procedure with standardized surgical equipment aiming to eliminate surgical variability [26].

Previous BCHI clinical trials used non-validated outcome measurement scales, creating a need for validated alternatives. Most questionnaires and endpoints currently used to evaluate the BCHI from the perspective of the clinicians rather than that of the patients’ [30]. We know that studies using non-validated scales are prone to risk of bias [31]. In contrast, studies that use reliable, systematic and validated outcome measures give the possibility to compare different trials and perform a meta-analysis [32]. Another limitation is that the number of prospective randomized controlled trials in the field is low [22, 33–36]. Moreover, complications related to the BCHI can vary over time and may be missed by only assessing patients at fixed time points coinciding with preplanned trial visits. Important information is lost this way and incidence numbers might become under- or overestimated. By not collecting all the information which is available, the ability to find a difference between two interventions also decreases. In this clinical trial, the amount of standard visits is decreased in favor of collecting more information during extra consultations in case of problems.

The decision to choose one intervention over the other in a health care system *with limited resources* depends on the associated (clinical) benefits, but also on the incurred additional costs or cost savings. As reviewed by Crowson et al. [4], only a few investigations addressed the cost-effectiveness of a BCHI intervention, resulting in uncertainty regarding cost-effectiveness. One retrospective study by Monksfield et al. has been executed in the United Kingdom that compared gain in quality-adjusted life years to BCHI-related costs. Costs included: implantation surgery, post-surgical care, the first processor, annual check-up, processor maintenance, and processor replacement costs after 3 years [5]. This study concluded that the BCHI is probably cost-effective. So far, no study included non-health care costs, such as loss of productivity, travel costs and out-of-pocket costs.

In this article, we describe the research protocol for a multicenter randomized controlled study comparing the new MIPS technique to the linear incision technique with soft tissue preservation [24, 26] comparing the incidence of inflammation as primary objective. In this trial, revised parameter scales are introduced to quantify factors such as loss of skin sensibility, dehiscence of the skin next to the abutment, skin overgrowth, and cosmetic results. These are modified to reduce the subjective interpretation and are intended to be validated. For exploratory outcome measures related to PAD such as skin biopsies, bacterial swabs are collected. An economical evaluation is planned with quality of life approached from a capability perspective as well. The MIPS technique is hypothesized to result in a lower incidence of inflammation compared to the linear incision technique with soft tissue preservation.

## Methods

### Study design, ethics, setting, and recruitment

This study is a sponsor-initiated multicenter, open, randomized, controlled clinical investigation. This article has been drafted following SPIRIT guidelines [37] (See Table 1 in Additional file 1). Three hospitals in the Netherlands are currently recruiting participants for this study: Maastricht University Medical Center (MUMC+), ZiekenhuisGroep Twente (ZGT) and Medisch Centrum Leeuwarden (MCL). MUMC+ is an academic teaching hospital. ZGT and MCL are general hospitals. This multicenter study is performed in accordance with the Declaration of Helsinki [38], has been approved by the ethics committee of MUMC+ (NL50072.068.14/METC141007) and has been registered at clinicaltrials.gov (trial number: NCT02438618). The local ethics committees of ZGT (Adviescommissie locale uitvoerbaarheid wetenschappelijk onderzoek) and MCL (Commissie Onderzoeksverklaring) approved the local execution of this trial. A total of 62 participants will be recruited at the ear, nose and throat outpatient departments by the (local) researchers. Patients are considered eligible for participation [22, 39] (I) if they will undergo unilateral BCHI surgery and (II) when they are ≥ 18 years of age. Participants will be excluded from participation in case of (I) a history of immunosuppressive disease, (II) usage of systemic immunosuppressive medication, (III) bilateral BCHI placement, (IV) relevant dermatological disease (e.g., psoriasis, severe eczema), (V) participation in other studies, and (VI) when no suitable site for a 4-mm-wide implantation during surgery is found. For inclusion, written informed consent will be provided from all participants (See Additional file 2).

**Table 1:**
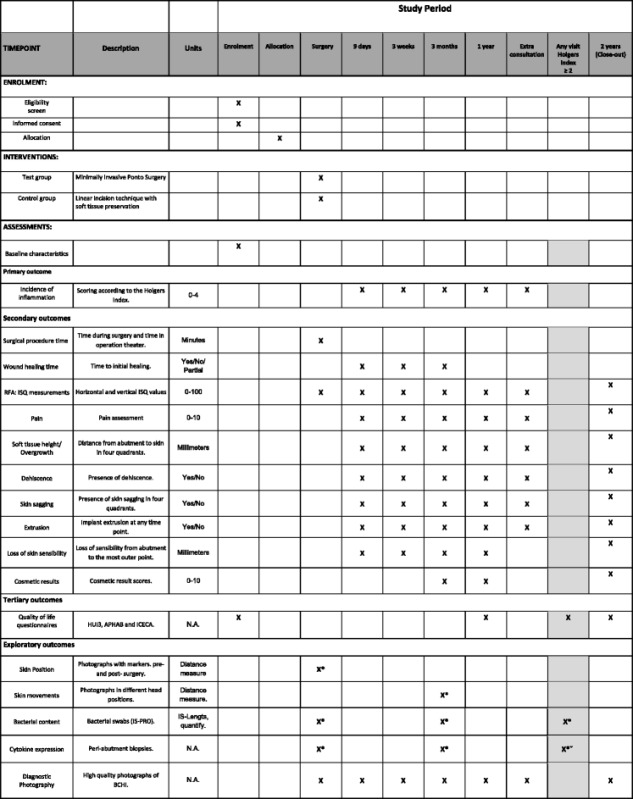
Schedule of enrolment, interventions, and assessments

Follow-up visits are planned post-surgery. Pain assessment: score for pain around the implant; radiating pain; headache associated with implant. Cosmetic result scores: score for natural skin position, baldness, scarring, skin color, indentation, overall cosmetic score. Light gray chart indicates measurements assessed at follow-up visits/extra consultations with a Holgers Index score ≥ 2

*RFA:ISQ* Resonance Frequency Analysis: Implant Stability Quotient, *HUI3* Health Utilities Index Mark 3, *APHAB* Abbreviated Profile of Hearing Aid Benefit, *ICECAP-A* ICEpop CAPability measure for Adults, *N.A*. not applicable, *IS-pro* a 16S-23S rDNA interspace (IS) region-based profiling method

^*^Outcome measurements obtained at Maastricht University Medical Center only

ˇOnly after explicit additional informed consent

### Study interventions and allocation

All participants will undergo single-stage surgery to receive a 4-mm Ponto wide implant with mounted abutment (Oticon Medical AB, Askim, Sweden), which will be performed by an experienced ENT surgeon. Four abutment lengths are available: 6, 9, 12 and 14 mm. Subjects will be randomized to the test group (MIPS technique [26]) or the control group (linear incision technique with soft tissue preservation [24]) in the order in which they enter the study. Skin thickness is a possible factor that could influence inflammation, but this is measured during surgery, making it impossible to stratify for skin thickness prior to surgery. Considering that men are known to have significantly thicker skin than women [40], group allocation is stratified for gender. Researchers will randomize each subject using randomization software (Statistiska Konsultgruppen, Gothenburg, Sweden), which will be performed in each research center independently in a 1:1 ratio for the test and control group stratified for gender until the total number of 62 participants has been reached. Due to clear differences in surgical techniques, it is impossible to blind the surgeon, researcher or subject. In short both surgical techniques are described here:

#### Control group (linear incision technique with soft tissue preservation) [24, 41]

**Fig. 1:**
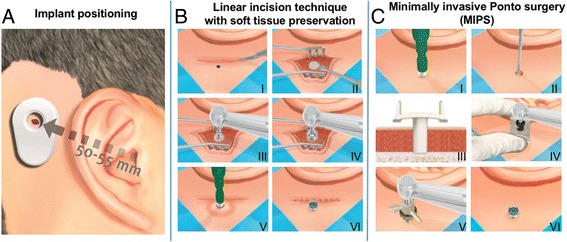
Surgical implantation techniques. **a** Implant positioning. **b** Schematic presentation of the linear incision technique with soft tissue preservation. (I) Linear incision. *(II)* Opening of skin. *(III)* Initial hole drilling. *(IV) C*ountersink drilling. *(V)* Eccentric skin punch to uncover abutment. *(VI)* Result. **c** Schematic presentation of Minimally Invasive Ponto Surgery (MIPS) technique. *(I)* Incision hole. *(II)* Removal of periost and soft tissue*. (III)* Placement of cannula. *(IV)* Drilling procedure (cannula guide drill and cannula widening drill). *(V)* Implant placement with the insertion indicator. *(VI)* Result

The intended implant position is marked (Fig. 1a). (I) A retroauricular linear incision is made down to the periosteum that is subsequently opened to expose the periosteum posterior to the incision line (Fig. 1b). (II) At the intended implant site, a central area of periosteum is removed. (III) An initial 3-mm-deep hole is created using a guide drill. (IV) If there is still bone at the bottom of the initial hole, it is deepened to 4 mm using the same drill by removing a spacer. (V) To prepare the initial hole for implant insertion it is widened with a countersink drill. (VI) The implant with mounted abutment is installed with a torque setting of 40–50 Ncm. (VII) The skin is retracted over the abutment and dermal sutures are placed. (VIII) The abutment is recovered by punching the skin with a 5-mm punch. (IX) A healing cap is attached to the abutment and gauze drenched in an antibiotic ointment is applied.

#### Test group (MIPS technique) [26, 42]

The intended implant position is marked (Fig. 1a). *(I)* An incision is created with a 5-mm punch at the intended implant site (Fig. 1c). *(II)* The periosteum and remaining soft tissue around the incision hole are removed with a raspatorium. *(III)* The cannula is inserted at the surgical site. *(IV)* A 3-mm hole is created initially with the cannula guide drill. *(V)* If there is bone at the bottom of the 3-mm hole, it is deepened to 4 mm using the same drill by removing a spacer*. (VI)* To prepare the initial hole for implant insertion, it is widened with the cannula widening drill. *(VII)* The cannula is removed and subsequently the implant with mounted abutment is installed with a torque setting of 40–50 Ncm*. (VIII)* To help estimate complete insertion of the implant, an installation indicator is attached to the abutment inserter, which makes it possible for the surgeon to count the number of rotations. This step was added during the course of the study. *(IX)* A healing cap is attached to the abutment and gauze drenched in an antibiotic ointment is applied.

### Follow-up

**Fig. 2:**
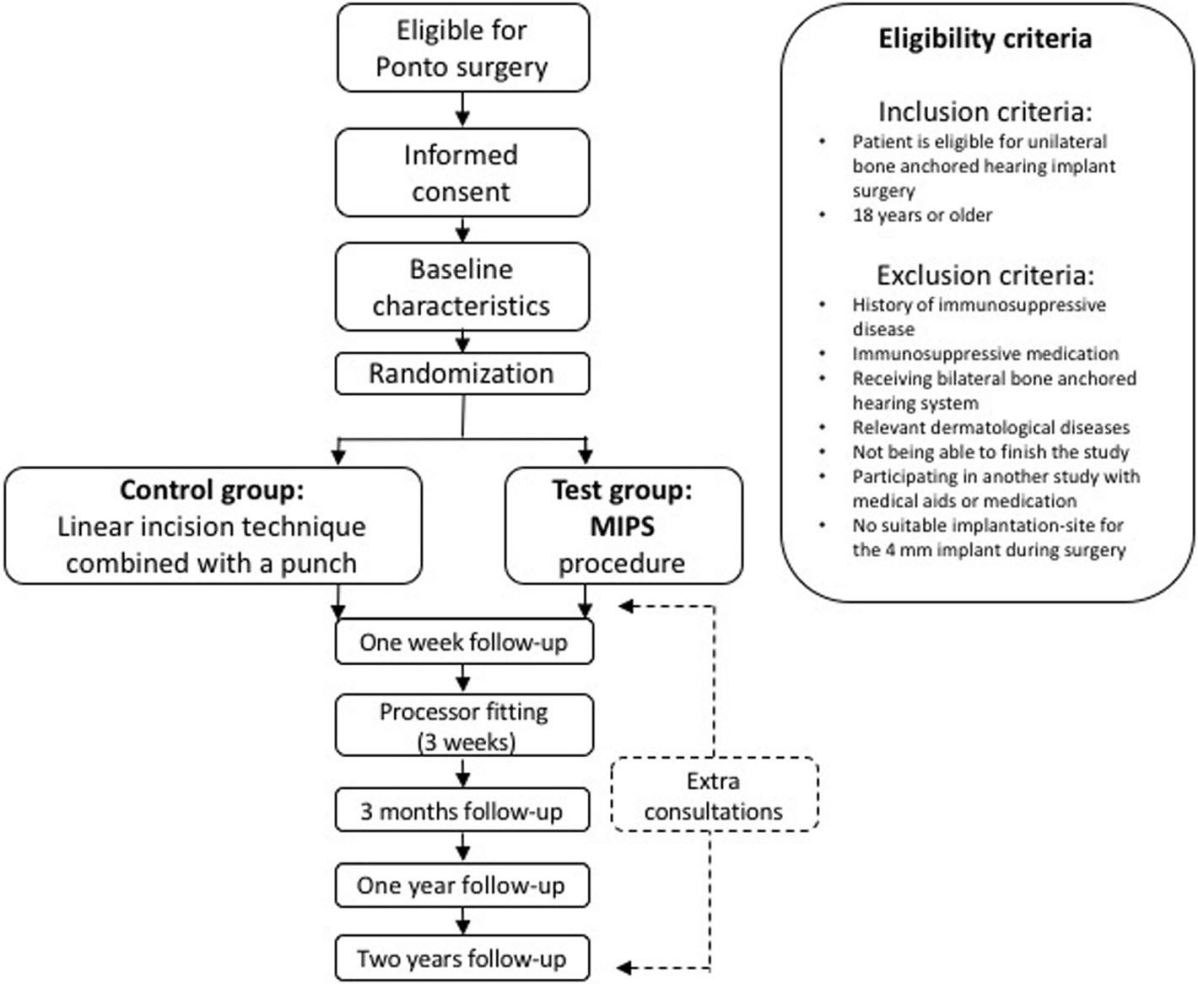
Study design and study flow chart

Follow-up visits for all participants are scheduled at 9 days, 21 days, 3 months, 1 year and 2 years post-surgery (Fig. 2). During this period all extra consultations are captured in high detail. The consultations are largely comparable to standard visits (Table 1). By compiling extra consultation visits and regular follow-up visits, we can present a more accurate estimate of common complications that can occur at any time point.

### Outcomes

#### Primary outcome

The primary outcome of this study is the incidence of inflammation (episodes of PAD) between surgery and 3 months post-surgery. During each visit, the peri-abutment skin is graded based on the Holgers Index. The Holgers Index is a five-point scale described by Holgers [11]: “0 no irritation; 1 slight redness; 2 red and slightly moist tissue, no granuloma formation; 3 reddish and moist; sometimes granulation tissue; 4 removal of skin-penetrating implant necessary due to infection” [11]. In this study, inflammation has been defined as the occurrence of a Holgers Index ≥ 2 as this often requires substantial treatment (e.g., systemic antibiotics or local intervention).

#### Secondary outcomes

Secondary outcomes include surgical procedure time, wound healing, presence of dehiscence after surgery, soft tissue height, loss of skin sensibility, pain, cosmetic results, implant stability quotient (ISQ) values, and extrusion rate (Table 1).

Surgical procedure time consists of the length of surgery (from the incision until the placement of the healing cap) and the total time spent in the operating theater by the subject (entering theater until leaving theater) in minutes. Both timings are measured using a stopwatch and are included because the preparation and post-surgical care can differ. Wound healing is evaluated during standard follow-up visits and can be graded as complete, partial or incomplete. The presence of dehiscence is evaluated at all visits. Soft tissue height and overgrowth is assessed by measuring the distance from the top of the abutment to the skin in four quadrants. Numbness is measured as the start of sensibility from the abutment to the most outward diameter. Pain is assessed for three separate domains including pain directly around the abutment, radiating pain, and headache that is related to the BCHI. Pain is graded in a 10-point scale with a scale of 0 representing “no pain” to 10 representing “the worst pain imaginable”. Cosmetic results are assessed by the surgeon using several properties which were thought to be influenced by the surgical technique. These included the folding of the skin around the abutment, baldness, scarring, skin color, indentation, and an overall cosmetic score as assessed by the surgeon and subject. All results are graded on a 10-point scale and compared to the contralateral side if applicable. ISQ values are obtained with the Ostell ISQ equipment (Ostell, Gothenburg, Sweden) by mounting a Smartpeg Type 55 on the abutment and obtaining two perpendicular ISQ values at all visits. All cases of abutment exchanges and implant extrusion are noted (time of extrusion, reason if known and subsequent action).

#### Tertiary outcomes

##### Quality of life and economic evaluation

Three questionnaires are used to evaluate the impact on hearing-specific and generic quality of life and capabilities. The questionnaires are filled in at baseline, at 1-year follow-up, and at 2-year follow-up. The Abbreviated Profile of Hearing Aid Benefit (APHAB) is a 24-item self-assessment, disability-based inventory, designed for hearing-related quality of life [43]. Each item is assessed in both the unaided and aided situation. The Health Utilities Index Mark 3 (HUI3) is a preference-based system for measuring generic health-related quality of life, consisting of 17 questions [44]. It provides descriptive evidence on multiple dimensions of health status, including overall health and several health dimensions. These dimensions include: vision, hearing, speech, ambulation/mobility, pain, dexterity, self-care, emotion, and cognition. Each dimension has three to six discriminatory levels, making it sensitive to health changes induced by interventions [45]. The ICEpop CAPability measure for Adults (ICECAP-A) was designed [46] to measure capabilities [47]. Capabilities represent the “freedom” of an individual to “achieve” a certain functioning, without the need to have actually achieved this [48]. This 5-point questionnaire has been translated and validated in Dutch [49]. It specifically assesses the capabilities attachment, security, enjoyment, role, and control. These questions include aspects such as independence, dignity, comfort, and social interaction, which might be influenced by hearing loss and subsequent interventions. A last tertiary objective of this study is a full economical evaluation after data has been collected for 1 year as we assume that 1 year after surgery, the complication rate, benefits, satisfaction, and BCHI processor usage are stable in both treatment groups. Cost will be identified at each study visit using the case report form (CRF), which is designed to identify costs. We will perform a cost-utility analysis, with the quality-adjusted life year calculated from the obtained HUI3 scores as the outcome. The analysis will be executed using a societal perspective. The evaluation will be performed according to the standards and guidelines of the International Society for Pharmacoeconomics and Outcomes Research (ISPOR) [50].

#### Exploratory outcomes

##### Skin displacement

High-resolution photographs (MUMC+: Nikon D800E, Nikon Corp., Tokyo, Japan) with an additional lens (Nikon AF-S VR Micro-Nikkon 105 mm f/2.8G IF-ED, Nikon Corp., Tokyo, Japan); ZGT and MCL: iPhone 6 (Apple Inc., Cupertino, CA, USA) are collected at surgery and follow-up visits. These photographs can be used to evaluate the peri-abutment skin over time and to assess the skin movability in relation to movements of the head and jaw using standardized skin markings. These photographs will also be used to study the validity of the Holgers Index and to investigate peri-abutment dermatitis. In addition, the minimal and maximal size of the gap between the abutment and skin is measured in all quadrants.

##### Etiology of peri-abutment dermatitis

These outcome measures are obtained at MUMC+ only. Skin biopsies of the implantation site are obtained during surgery, at 3 months post-surgery, and during episodes of inflammation if participants have explicitly provided an additional written informed consent for this procedure. RNA will be extracted from the biopsies and subsequently cDNA will be synthesized. We intend to determine mRNA expression of 15 selected genes (Table 2) using real-time reverse transcription polymerase chain reaction. Pre-surgical mRNA expression (baseline) and post-surgical expression will be compared. Specific profiles related to inflammatory responses, tissue remodeling, vascularization, and bacterial infection will be assessed.

**Table 2:** Overview of cytokines

Inflammatory mediators	TGF- ß MIP-1α	Microbial infection
IL-1ß		TLR-2
IL-6	Tissue remodeling	TLR-4
IL-8	MMP-9	
TNF-α	TIMP-1	Vascularization
IL-17	COL1 α1	VEGF-A
IL-10		FGF-2

*IL* interleukin, *TNF-α* tumor necrosis factor alpha, *TGF-ß* transforming growth factor beta, *MIP-1 α* macrophage inflammatory protein 1 alpha, *MMP-9* matrix metalloproteinase 9, *TIMP-1* tissue inhibitor of metalloproteinase 1, *COL1 α1* collagen, type 1, alpha 1, *TLR* Toll-like receptor, *VEGF-A* vascular endothelial growth factor A, *FGF-2* basic fibroblast growth factor-2

Bacterial swabs of the abutment, peri-abutment skin and contralateral skin are collected during the same time points. The bacterial content will be evaluated with IS-pro, a novel 16S-23S rDNA interspace (IS) region-based profiling method [51]. This method is devised to enable high-throughput molecular profiling of any microbiota. The combined data sets will be used to study peri-abutment dermatitis and validate the Holgers Index.

### Analysis

#### Sample size calculation

The incidence of inflammation, a Holgers Index ≥ 2, between surgery and 3 months post-surgery will be compared between both groups. The sample size is calculated using the concept of effect size (ES) as presented by Lerman and Cohen [52, 53]. The proposed test concerns a two-sample test for binomial proportions, which is equivalent to a Yates corrected chi-square test for a 2 × 2 contingency table. When taking into account a type 1 error level (α) of 0.05 and a power (1-β) of 0.8, the required sample size per group is: *n = 2(zα + zβ)* ^*2*^ */ES* ^*2*^ *= 2(1.65 + 0.84)* ^*2*^ */ES* ^*2*^ *= 12.4/ES* ^*2*^ *.* Assuming an ES index of *medium to large* with ‘h’ of 0.65 to discriminate between two proportions, the sample size is: *n = 12.4/h* ^*2*^ *= 12.4/0.65* ^*2*^ *= 29.4*. With an expected 5 % drop-out rate, 31 participants are needed per group, resulting in a total sample size of 62 participants.

#### Statistical analysis

This study is designed to include several endpoints including short-term results (3 months follow-up), long-term results (2 years follow-up), and an economic evaluation. Short-term results will be evaluated after all participants have reached 3 months follow-up and will describe the primary endpoint and secondary endpoints between surgery and 3 months follow-up. The primary endpoint will be described by comparing the proportions of inflammation between surgery and 3 months follow-up using a chi-square test. Long-term results will also be used for the economic analysis. Prior to analysis, a statistical analysis plan (SAP) will be created describing the method of analysis specifically for each endpoint.

### Safety

Cases of adverse events or device deficiencies will be recorded in the CRF. All cases of serious adverse events (SAE) will be recorded in the corresponding CRF as well and subsequently reported to the sponsor and responsible regulatory committees. The recorded events will be incorporated in the applicable study results.

### Study management, oversight and publication

The study is monitored by Oticon Medical AB in conjunction with TFS (Zaltbommel, The Netherlands). Due to the low risk classification of the investigated procedures, a data monitoring committee was not deemed necessary. Data handling will conform to Dutch legislation. Source data is contained in the original records (the electronic patient dossier), original forms (questionnaires), and CRF. Data will be kept for 15 years. A data management team, consisting of the principal investigator, researchers from the coordinating center, and representatives from the sponsor are established to oversee and manage data collection. Any collected human tissue will be adequately disposed of after analysis. Insurances are provided for all participants in accordance with Dutch legislation. Regular care will continue if a subject has finished follow-up or if a participant withdraws from the study. The results of this study will be submitted to peer-reviewed journals without any publication restrictions by the sponsor.

### Protocol amendments

After initial approval (protocol version 1.2 dated 4 November 2014), the study site opened for accrual on 1 December 2014. So far, the ethics committee has approved two substantial amendments to the study protocol. In June 2015, an amendment (protocol version 1.4 12 May 2015) was approved to increase the total number of participants from 42 to 62, resulting in a decrease of the ES from 0.8 to 0.65 with 80 % power to discriminate between two proportions. In the same amendment, in order to include sufficient participants, the study was expanded from a single-center study to a multicenter study. In October 2015, the second amendment (protocol version 1.5 11 August 2015) was approved to include MIPS surgical equipment update, based on feedback after the first surgeries. The alterations in MIPS surgical package included shortening of the cannula and the addition of wings in order to increase the grip and stability of the cannula. The guide drill was also modified, resulting in a wider drilling hole making it easier to manually feel the drilling hole with the widening drill. An installation indicator was also developed, to assist in visual feedback for insertion completeness by counting the number of rotations during insertion. These updates were incorporated as MIPS became available to multiple centers around the world and surgeons provided feedback in relation to their individual outcomes. These updates are not expected to affect the primary endpoint in a significant way, hence no change in the number of participants was necessary for this amendment. The change in the surgical procedure might influence secondary outcome measures (e.g., surgery time, risks on complications such as incomplete insertion) to a limited extent. All MIPS surgeries will be analyzed as one pooled treatment group but the number of participants per MIPS version will be reported on.

## Discussion

In the design of this prospective multicenter trial, a total of 62 participants are intended to be included and followed for a period of 2 years. In this trial, the MIPS procedure (designed to reduce tissue trauma, standardize surgery, and alleviate the need for an incisional scar [26]) is compared to the soft tissue preservation technique [23, 24], which can be regarded as the conventional method. The incidence of PAD (Holgers Index ≥ 2) between surgery and 3 months follow-up is the primary outcome measurement. Adapted measures scales describing loss of skin sensibility, cosmetic results, and skin height are introduced. Additionally, various exploratory measurements including skin biopsies, bacterial profile, and skin movement are incorporated in this study. This exploratory data might allow conducting of correlative and comparative analyses between the onset of PAD, a changed gene expression, and the presence of different bacterial species. This might help in understanding the multifactorial role bacteria, the immune response, and skin movements play in the etiology of PAD.

One of the objectives of this investigation is to collect biopsies and photographs to explore the incidences of inflammation. The latter will also be used to validate the Holgers Index or, if that is not achieved, to create a new scale for which an internal validation will then be available. Newly introduced outcome measures such as loss of sensibility, cosmetic outcomes, pocket size, skin height, and skin sagging have not yet been validated. These scales are designed to include as little as possible subjective interpretation (e.g., using millimeters and predefined quadrants). Also because every subject will be photographed at every visit, the interpretation of the Holgers Index score can be justified post hoc and the validity can be further investigated in the future.

Another important aspect this study addresses is the timing and frequency of relevant information sampling. The occurrence of complications may be missed while following the standardized time points (e.g., patients do not only show infections at predetermined visits). Variable sampling over time will possibly allow for a more accurate assessment of the duration and incidences of complications such as PAD, sensibility loss, and pain over time. The use of areas under the curve (AUC) is proposed here as a solution for incorporating information available from these extra consultations. We assume that and encourage participants to visit their ENT surgeon if they experience a complication (e.g., inflammation or pain). By incorporating episodes of complications, the complication and its burden can be assessed over time. This approach has been estimated to result in an increased power without having to increase subject numbers. In addition, a more accurate representation of complications over time can possibly be achieved.

Besides conventional and hearing-specific quality-of-life approaches (e.g., HUI3 and APHAB) this study also uses the ICECAP questionnaire that focuses on the wellbeing of a participant in a broad perspective. In the field of BCHI and related technologies, it is plausible that interventions have more benefits than just health gains or hearing improvements. These devices and interventions might also increase the autonomy, freedom to achieve or develop, and impact on social interaction. These factors are important in the perspective of a person as a whole [54–56] and are currently overlooked by most questionnaires. Potentially, these factors may be playing an important role in cost-utility or effectiveness. Moreover, BCHI recipients consist of a diverse complex population that might broadly differ in experienced benefit (e.g., SSD subjects [57]). Arguably, it might turn out that the ICECAP adds an important new dimension to consider with the availability of transcutaneous solutions as well. This might add a new perspective to the current complexity of the decision-making process surrounding BCHI placement (many different device choices with highly different profiles), reimbursement policies, complications, and patient preferences.

### Limitations

Due to the differences in surgical procedures it is impossible to perform a blinded trial. The surgical wound created is evident for the allocated intervention to the clinician and the patient. The new intervention could potentially lead to unexpected events, as both the surgical approach and surgical tools have been modified extensively. To identify possible issues, (serious) adverse events will be qualitatively assessed to allow for a correct identification of differences and potential drawbacks. Although several methods have been implemented to maximize the collection of information on trial participants to increase the power of this trial, the small sample size in this study remains to be a major drawback.

## Trial status

Recruitment started in December 2014 and is currently still ongoing. The predicted study completion date is August 2018. Short-term results are expected in the last quarter of 2016.

## Additional files

Additional file 1: SPIRIT checklist. (PDF 76 kb)
Additional file 2: Subject information. (PDF 159 kb)

## Abbreviations

APHAB: Abbreviated profile of hearing aid benefit
AUC: Area under the curve
BAHA: Bone-anchored hearing aid
BCHI: Bone conduction hearing implant
CRF: Case report form
ES: effect size
HL: Hearing loss
HUI3: Health utilities index mark 3
ICECAP-A: ICEpop CAPability measure for Adults
ISQ: Implant stability quotient
MCL: Medisch centrum Leeuwarden
MIPS: Minimally invasive ponto surgery
MUMC+: Maastricht University Medical Center
PAD: Peri-abutment dermatitis
SAE: Serious adverse event
SSD: Single-sided deafness
ZGT: ZiekenhuisGroep twente
