# Supplementary material for: Minimally Invasive Ponto Surgery compared to the linear incision technique without soft tissue reduction for bone conduction hearing implants: study protocol for a randomized controlled trial
Source: Trials. 2016 Nov 9;17:540. doi: 10.1186/s13063-016-1662-0 (PMC5103483; doi:10.1186/s13063-016-1662-0)
Supplement: Additional file 2: — Subject information. (PDF 159 kb) [file 13063_2016_1662_MOESM2_ESM.pdf]

Informatiebrief (versie 1.5 13-01-2016)

Minimaal invasieve chirurgie voor Ponto botverankerde hoortoestellen

Geachte heer/mevrouw,

Wij vragen u vriendelijk om mee te doen aan een medisch-wetenschappelijk onderzoek (zie titel). U komt in aanmerking voor deelname omdat u een botverankerd hoortoestel, het Ponto® systeem ontvangt. U beslist zelf of u wilt meedoen. Voordat u de beslissing neemt, is het belangrijk om meer te weten over het onderzoek. Lees deze informatiebrief rustig door. Bespreek het met uw partner, vrienden of familie. Lees ook de Algemene brochure. Daar staat veel algemene informatie over medisch-wetenschappelijk onderzoek in. Het onderzoek wordt hier uitgevoerd (Afdeling KNO, Academisch Ziekenhuis, Maastricht) onder leiding van hoofdonderzoeker Prof. dr. Stokroos. Het onderzoek wordt gesponsord door de fabrikant van het botverankerd hoortoestel, Oticon medical AB in Askim, Zweden.

1. Wat is het doel van het onderzoek?

Wij onderzoeken of een nieuwe operatietechniek voor het plaatsen van een Oticon Medical Ponto systeem tot betere resultaten leidt. Het Ponto systeem zelf zal voor iedereen gelijk zijn. Het botverankerd hoortoestel wordt niet onderzocht in dit onderzoek. De fabrikant kan de kennis opgedaan in het onderzoek toepassen om de nieuwe operatie voor alle patiënten in de toekomst te adviseren. Na de operatie zullen we kijken of het optreden van problemen minder vaak voorkomt rond het botverankerd hoortoestel. We kijken dan naar dingen als pijn, infecties, een verminderd gevoel rond het botverankerd hoortoestel, huidovergroei rond het botverankerd hoortoestel, kwaliteit van leven en de kosten die u en het ziekenhuis heeft gemaakt. We zullen ook kijken hoe het botverankerd hoortoestel er uiteindelijk uit ziet als het bij u geplaatst is. Daarnaast onderzoeken we de huid die rondom het botverankerd hoortoestel ligt. Dit doen we door foto’s te maken. We bekijken ook welke bacteriën zich hier nestelen en of de huid daar geïrriteerd of ontstoken van raakt. We vergelijken dit dan met de situatie voor de operatie. Daarmee hopen we een idee krijgen hoe we deze ontstekingen het beste kunnen behandelen of voorkomen. We doen dit door tijdens de operatie de huid te verzamelen op de plaats waar uw Ponto komt te zitten. Normaal zou deze huid worden weggenomen. Nu wordt

deze bewaard voor het onderzoek. Na de operatie herhalen we de meting door met een wattenstaafje wat bacteriën te verzamelen rondom het botverankerd hoortoestel. Daar voelt u niets van. Ook nemen we met een holle naald wat weefsel af. Dit is vergelijkbaar met een bloedafname. We zullen de huid verdoven zodat u hier zo min mogelijk last van heeft. Dit is een vrijwillig onderdeel van het onderzoek. U kunt ervoor kiezen om niet mee te doen met dit onderdeel van het onderzoek. In de toekomst hopen we met de kennis die we met dit onderzoek opdoen bijwerkingen en ontstekingen sneller kunnen opsporen en beter kunnen behandelen.

2. Welke product en behandeling worden onderzocht?

U bent reeds ingelicht over het Ponto systeem. Indien u hier nog vragen over heeft, kunt u deze stellen aan uw behandelend arts. De huidige operatietechniek waar al veel ervaring mee is wordt vergeleken met een nieuwe operatietechniek in dit onderzoek. De nieuwe operatietechniek is op enkele punten aangepast. In dit onderzoek kijken we of dit dan ook tot betere resultaten leidt. We verwachten geen grote verschillen. Omdat we met de fabrikant samenwerken kan een kleine verbetering al tot veel winst voor alle nieuwe Ponto gebruikers leiden. Als u nog vragen heeft over de details van de verschillende operatietechnieken kunt u dit bij de onderzoeker of uw KNO-arts aangeven. Deze zal het dan voor u tekenen en beschrijven.

3. Hoe wordt het onderzoek uitgevoerd?

Dit onderzoek is multi-centrum onderzoek. In totaal zullen 62 patiënten meedoen. De helft van de patiënten zal geopereerd worden met de nieuwe operatietechniek. De andere helft zal met de traditionele operatietechniek worden behandeld. Als u besluit deel te nemen zal u door loting in één van de twee groepen ingedeeld worden. Bij deelname kunt u dus niet zelf beslissen welke operatietechniek u zult ondergaan. Indien u niet meedoet wordt u met de traditionele operatietechniek behandeld.

4. Wat wordt er van u verwacht?

Voor het onderzoek moet u voornamelijk vragenlijsten in vullen, vragen van de arts beantwoorden en metingen aan de Ponto ondergaan tijdens de reguliere controle afspraken gedurende een periode van 2 jaar. Dit kost extra tijd, ongeveer 30 minuten per keer dat u komt. Deze controles doen geen enkele pijn. We maken ook foto's van uw Ponto systeem. Deze zijn niet tot u te herleiden. Het kan zijn dat er in verhouding tot de standaard zorg extra bezoeken nodig zijn voor het onderzoek. Daarnaast willen we het stukje huid dat wordt verwijderd voor het plaatsen van het Ponto-systeem tijdens de operatie graag bewaren voor het onderzoek.

Ook vragen we u of we wat weefsel met een holle naald mogen afnemen rondom de Ponto. Dit is helemaal vrijwillig. U kunt er ook voor kiezen aan het onderzoek mee te doen zonder dat we wat weefsel afnemen. We vragen u het daarom apart. Bijvoorbeeld als u het belastend vindt dat we dit doen. We doen dit op de polikliniek met een holle naald met een doorsnede van 1 millimeter. Als we dit doen zullen we het gebied eerst verdoven, zodat u er zo min mogelijk last van heeft. De naald van de verdoving kan pijnlijk zijn. Ook kan de naald voor een kleine bloeding zorgen.

5. Wat is meer of anders dan de reguliere behandeling die u krijgt?

U wordt per loting ingedeeld in een van de twee groepen. U kunt hierbij worden ingedeeld in de groep die wordt behandeld met de nieuwe operatietechniek of in de groep die wordt behandeld met de traditionele techniek. De patiënten in dit onderzoek worden gelijk verdeeld over de twee groepen. We besteden alleen meer tijd aan het noteren en observeren van hoe het met u en uw Ponto systeem gaat. Het kan zijn dat u meer controle afspraken heeft dan dat u normaal zou hebben. In dat geval vergoeden we de reiskosten voor de extra bezoeken.

Als u deelneemt aan het onderzoek hebben we een aantal extra metingen, die u normaal niet zou ondergaan. We vragen u een aantal keer om vragenlijsten in te vullen. We meten elk bezoek hoe vast uw botverankerd hoortoestel in uw bot zit. Dit is een pijnloze en snelle meting. We gaan een aantal keer met een borstel langs uw bortverankerd hoortoestel om te kijken welke bacteriën er zitten. Ook dit is pijnloos. Tijdens al uw bezoeken maken we een

foto van uw botverankerd hoortoestel. We zullen ervoor zorgen dat u niet herkent kunt worden op deze foto`s.

Tijdens de operatie nemen we een stukje huid af waar het botverankerd hoortoestelkomt. Normaal wordt dit weggegooid, nu bewaren we dit. Als extra optie kunt u ervoor kiezen dat we wat weefsel rondom de Ponto mogen afnemen. Dit doen we dan na 3 maanden en als u een ontsteking zou krijgen. U kunt er dan telkens voor kiezen om hier van af te zien. We kunnen dan kijken of de ontstekingswaarde in dit verwijderde materiaal een indicatie is voor de ernst van de ontsteking. Deze methode kan dan in een later stadium gebruikt worden om het botverankerd hoortoestel of de behandeling van bijwerkingen te verbeteren.

6. Welke bijwerkingen kunt u verwachten?

We voorzien geen extra complicaties of risico's. De standaard Ponto plaatsing heeft natuurlijk wel risico's. We herhalen ze hier. Er bestaat het risico op de volgende complicaties in de regio rondom het botverankerd hoortoestel:

- Infectie en verdikking van de huid (1-20%)
- Pijn (<5%)
- Gevoelloosheid (<5%)
- Het verlies van het botverankerd hoortoestel (<5%)

Deze bijwerkingen kunnen te allen tijde ontstaan. Mocht u problemen ondervinden of een bijwerking hebben die hier niet vermeld staat, moet u contact opnemen met de polikliniek KNO in het Academisch ziekenhuis Maastricht. U kunt daar vragen naar uw behandelend KNO-arts of naar de onderzoeker, drs. T. Calon. Vermeld altijd dat u aan het Ponto onderzoek meewerkt. Als er sprake is van een ernstige situatie buiten kantoor uren kunt u contact opnemen met de Spoedeisende Hulp in het Academisch ziekenhuis Maastricht.

Indien we ook een kleine hoeveelheid weefsel mogen opzuigen met een holle naald brengt dit wel een aanvullend risico mee, namelijk dat het gaat bloeden, er een blauwe plek ontstaat of dat het een ontsteking geeft. Dit verwachten we niet. U kunt in dat geval met ons contact opnemen. We kunnen dit over het algemeen goed behandelen met een antibiotica zalf of een

pleister. De afname zelf kan u lokaal tijdelijke pijnklachten geven. We zullen altijd een lokale verdoving zetten. De pijn is beperkt tot het zetten van de lokale verdoving.

7. Wat zijn mogelijke voor- en nadelen van deelname aan dit onderzoek?

We verwachten dat de risico's voor beide technieken vergelijkbaar zijn. Mogelijk heeft deze nieuwe operatietechniek betere resultaten. Onderzocht zal worden of de deelnemers die ingedeeld worden in de groep met de nieuwe operatietechniek een mooier litteken hebben, een kortere genezingstijd of minder complicaties ervaren. Het nadeel aan deelname is de extra tijd die het u kost. Het is mogelijk dat er met deze nieuwe techniek meer complicaties optreden.

Het nadeel van het afnemen van weefsel is met name pijn. De risico's hiervan zijn vergelijkbaar met het afnemen van bloed. Dat zijn lokale pijn, een bloeding, een blauwe plek en een lokale ontsteking. We zullen de pijn beperken door een lokale verdoving te geven.

8. Wat gebeurt er als u niet wenst deel te nemen aan dit onderzoek?

U beslist zelf of u meedoet aan het onderzoek. Deelname is vrijwillig. Als u besluit niet mee te doen, hoeft u verder niets te doen. U hoeft niets te tekenen. U hoeft ook niet te zeggen waarom u niet wilt meedoen. U krijgt gewoon de behandeling die u anders ook zou krijgen. Als u wel meedoet, kunt u zich altijd bedenken en toch stoppen. Ook tijdens het onderzoek.

9. Wat gebeurt er als het onderzoek is afgelopen?

Indien het eind van de studie bereikt is, zal uw controle en eventuele behandeling doorgezet worden in het kader van reguliere zorg. Er zijn voor u geen risico's verbonden aan het tussentijds stoppen met het onderzoek. Indien het onderzoek door gegronde redenen eerder gestaakt wordt dan gepland zal u hierover bericht worden.

10. Bent u verzekerd wanneer u aan het onderzoek meedoet?

Voor iedereen die meedoet aan dit onderzoek is een verzekering afgesloten. De verzekering dekt schade als gevolg van het onderzoek. Dit geldt voor schade die naar boven komt tijdens het onderzoek, of binnen vier jaar na het einde van het onderzoek. In de bijlage Verzekering vindt u de verzekerde bedragen, de uitzonderingen en de adresgegevens van de verzekeraar.

11. Wordt u geïnformeerd als er tussentijds voor u relevante informatie over de studie bekend wordt?

Het onderzoek zal zo nauwkeurig mogelijk volgens plan verlopen. Maar de situatie kan veranderen. Als dat zo is, bespreken we dat direct met u. U beslist dan zelf of u met het onderzoek wilt stoppen of doorgaan. Als uw veiligheid of welbevinden in gevaar is, stoppen we direct met het onderzoek.

12. Wat gebeurt er met uw gegevens?

In dit onderzoek zal er met de gegevens omgegaan worden zoals dat ook beschreven is in de bijgevoegde Algemene Brochure Medisch-Wetenschappelijk Onderzoek met Mensen.
Wij zijn verplicht uw gegevens te bewaren gedurende 15 jaar. Mocht u hier niet mee akkoord zijn dan kunt u niet aan het onderzoek mee doen. Gegevens die we van u bewaren bevatten persoonsgegevens, de resultaten van het onderzoek en gegevens over uw gezondheidstoestand. Als we de gegevens nogmaals willen gebruiken vragen wij u opnieuw om uw toestemming. U kunt dan nog beslissen of wij uw gegevens echt mogen gebruiken.
U kunt ten alle tijden uw toestemming om uw gegevens te gebruiken in trekken. Dit kunt u doen door dit schriftelijk kenbaar te maken aan onderzoeker drs. T. Calon. Er zullen dan geen nieuwe gegevens meer verzameld worden. U heeft het recht op inzage in uw gegevens. U kunt ook hiervoor contact opnemen met drs. T. Calon. De fabrikant van de Ponto (Oticon medical) kan anonieme gegevens over uw gezondheidstoestand en de foto’s van uw botverankerd hoortoestel van Nederland naar andere landen overdragen. Bijvoorbeeld in een presentatie over het nieuwe product. Mogelijk dat de privacy wetten daar minder strikt zijn dan hier. Omdat de gegevens anoniem zijn, zullen deze nooit herleidbaar zijn naar u. Indien er herkenbare punten op de foto zouden staan dan worden deze onherkenbaar gemaakt. U dient hier wel toestemming voor te geven. Het afgenomen weefsel zal worden geanalyseerd waarna het wordt vernietigd.

13. Wordt uw huisarts en/of behandelend specialist geïnformeerd bij deelname?

Wij laten uw huisarts schriftelijk weten dat u meedoet aan het onderzoek. Ook schrijven we dit op in uw patiëntendossier. Zo is ook de afdeling KNO en uw behandelend KNO arts op de hoogte van uw deelname. Dit is voor uw eigen veiligheid. U moet hiervoor toestemming geven op het toestemmingsformulier. Als u geen toestemming geeft, kunt u niet meedoen aan het onderzoek.

14. Zijn er extra kosten/is er een vergoeding wanneer u besluit aan dit onderzoek mee te doen?

Wij vergoeden uw gemaakte reiskosten als u door het onderzoek extra naar het ziekenhuis moet komen. De reiskosten moeten wel redelijk zijn en in verhouding staan met de afstand die u moet reizen. Ook moet u hiervoor een afschrift inleveren.

15. Welke medisch-ethische toetsingscommissie heeft dit onderzoek goedgekeurd?

De Toetsingscommissie van het AZM en de Universiteit Maastricht heeft dit onderzoek goedgekeurd. Meer informatie over de goedkeuring vindt u in de Algemene brochure.

16. Wilt u verder nog iets weten?

U kunt wat tijd nemen om een beslissing te maken als u dat wenst. U heeft altijd recht op een week bedenktijd. Geeft u dan bij uw volgende bezoek aan of u wel of niet mee wilt doen. Voor alle vragen kunt u terecht bij de onderzoeker drs. Tim Calon (043-3872842) of hoofdonderzoeker professor dr. R. J. Stokroos (te bereiken via de polikliniek 043-3875700). Wilt u graag een onafhankelijk advies over meedoen aan dit onderzoek? Dan kunt u terecht bij een onafhankelijke arts van onze afdeling, dr. L.W.J. Baijens (te bereiken via de polikliniek 043-3875700). U kunt met hem een telefonische afspraak maken via onze polikliniek Keel-, Neus- en Oorheelkunde.

17. Bijlagen

Bijgevoegd zijn de Algemene Brochure medisch-wetenschappelijk onderzoek met mensen en de verzekeringstekst.

Contactgegevens

Hoofdonderzoeker Academisch ziekenhuis Maastricht:
Professor dr. R. J. Stokroos

Polikliniek Keel-, Neus- en Oorheelkunde
Telefoon: 043-3875700

Onderzoeker:

Drs. T. Calon
Promovendus KNO

Polikliniek Keel-, Neus- en Oorheelkunde
Academisch ziekenhuis Maastricht
P. Debyelaan 25, 6229 HX Maastricht

Onafhankelijk arts:

Dr. L.W.J. Baijens
KNO-arts

Polikliniek Keel-, Neus- en Oorheelkunde
Academisch ziekenhuis Maastricht
P. Debyelaan 25, 6229 HX Maastricht
Telefoon: 043-3875700

Ondertekening toestemmingsformulier C50 studie

Minimaal invasieve chirurgie voor Ponto botverankerde hoortoestellen

NL50072.068.14 Versie 1.0 14.07.2014

Ik heb de informatiebrief voor de proefpersoon gelezen. Ik kon aanvullende vragen stellen. Mijn vragen zijn genoeg beantwoord. Ik had genoeg tijd om te beslissen of ik meedoe.

Ik weet dat meedoen helemaal vrijwillig is. Ik weet dat ik op ieder moment kan beslissen om toch niet mee te doen. Daarvoor hoef ik geen reden te geven.

Ik geef toestemming om mijn huisarts te vertellen dat ik meedoe aan dit onderzoek.

Ik geef toestemming om de specialist(en) die mij behandelt te vertellen dat ik meedoe aan dit onderzoek.

Ik weet dat sommige mensen mijn gegevens kunnen zien. Die mensen staan vermeld in de Algemene brochure.

Ik geef toestemming om mijn gegevens te gebruiken, voor de doelen die in de informatiebrief staan.

*Ik geef *wel/geen* toestemming om mijn gegevens te gebruiken voor onderzoek in de lijn van dit onderzoek.

* Ik geef *wel/geen* toestemming mijn afgenomen weefsel tijdens de operatie te gebruiken voor het onderzoek zoals beschreven in de informatiebrief.

Ik geef toestemming om gegevens nog maximaal 15 jaar na afloop van dit onderzoek te bewaren.

Ik geef toestemming dat de sponsor van het onderzoek (Oticon) de anonieme verzamelde gegevens over mij en foto’s van mijn botverankerd hoortoestel binnen of buiten de Europese

Unie verplaatst en gebruikt in publicaties beoogd op wetenschap of marketing.

Ik vind het goed om aan dit onderzoek mee te doen en zal de instructies van de onderzoeker opvolgen.

* Ik geef *wel/geen* toestemming om benaderd te worden voor eventueel toekomstig onderzoek

* Ik wens *wel/niet* geïnformeerd te worden over de resultaten van de studie

* Ik geef *wel/geen* toestemming dat de onderzoeker bij mijn botverankerd hoortoestel weefsel afneemt rondom mijn Ponto tijdens een vervolgbezoek.

Naam proefpersoon:

Handtekening: Datum : __ / __ / __

Ik verklaar hierbij dat ik deze proefpersoon volledig heb geïnformeerd over het genoemde onderzoek.

Als er tijdens het onderzoek informatie bekend wordt die de toestemming van de proefpersoon zou kunnen beïnvloeden, dan breng ik hem/haar daarvan tijdig op de hoogte.

Naam onderzoeker (of diens vertegenwoordiger):

Handtekening: Datum: __ / __ / __

Aanvullende informatie is gegeven door (indien van toepassing):

Naam: Functie:

Handtekening: Datum: __ / __ / __

---

* Doorhalen wat niet van toepassing is

Bijlagen

INFORMATIE OVER DE VERZEKERING

Voor iedereen die meedoet aan dit onderzoek is een verzekering afgesloten. De verzekering dekt schade, die het gevolg is van deelname aan het onderzoek. Dit geldt voor schade die naar boven komt tijdens het onderzoek, of binnen vier jaar na het einde van het onderzoek. U moet de schade ook binnen die vier jaar aan de verzekeraar hebben gemeld.

In geval van schade kunt u zich direct wenden tot de verzekeraar of schaderegelaar.

mw. mr. I. Speetjens of mw. mr. A. Frijlink
Academisch ziekenhuis Maastricht
Postbus 5800 6202 AZ Maastricht
Tel. 043-3875994 of 043-3875981

De verzekeraar van het onderzoek is:
Naam: Lloyd's London
Adres: Marketform Holdings Ltd, London EC 3M5EA
Telefoonnummer: Tel. 0044 1712201718

De schaderegelaar van het onderzoek is:
Naam: Dhr. A.D. Fontein
Adres Debussystraat 2, 3161 WD Rhoon
Telefoonnummer: 010-5066600

De verzekering biedt een maximumdekking van € 450.000 per proefpersoon en € 3.500.000 voor het gehele onderzoek en € 5.000.000 per jaar voor alle onderzoeken van dezelfde opdrachtgever. De dekking van specifieke schades en kosten is verder tot bepaalde bedragen beperkt. Deze vindt u in het Besluit verplichte verzekering bij medisch-wetenschappelijk onderzoek met mensen. Informatie hierover kunt u vinden op de website van de Centrale Commissie Mensgebonden Onderzoek: www.ccmo.nl.

Voor deze verzekering gelden een aantal uitsluitingen. De verzekering dekt de volgende schade niet:

- schade waarvan op grond van de aard van het onderzoek (nagenoeg) zeker was dat deze zich zou voordoen;
- schade aan de gezondheid die ook zou zijn ontstaan als u niet aan het onderzoek had deelgenomen;
- schade die het gevolg is van het niet (volledig) nakomen van aanwijzingen of instructies;
- schade aan nakomelingen, als gevolg van een nadelige inwerking van het onderzoek op u of uw nakomeling;
- bij onderzoek naar bestaande behandelmethoden: schade die het gevolg is van één van deze behandelmethoden;
- bij onderzoek naar de behandeling van specifieke gezondheidsproblemen: schade die het gevolg is van uitblijvende verbetering of van verslechtering van deze gezondheidsproblemen.
